# Extracellular Signal-Regulated Kinase and Reactive Oxygen Species Regulate PD-L1 to Promote Migration and Proliferation of Triple-Negative Breast Cancer MDA-MB-231 Cells

**DOI:** 10.32604/or.2026.077693

**Published:** 2026-07-16

**Authors:** Ching-Chun Ho, Yen-Cheng Chen, Wei-Liang Lean, Wen-Sheng Wu

**Affiliations:** 1 Division of General Surgery, Department of Surgery, Hualien Tzu Chi Hospital, Buddhist Tzu Chi Medical Foundation, Hualien, Taiwan; 2 School of Medicine, Tzu Chi University, Hualien, Taiwan

**Keywords:** Triple-negative breast cancer, programmed death-ligand 1, extracellular signal-regulated kinase, reactive oxygen species, signal transduction, targeted therapy

## Abstract

**Objectives:** Triple-negative breast cancer (TNBC) is a highly aggressive form of breast cancer. Mitogen-activated protein kinases (MAPKs), including extracellular signal-regulated kinase (ERK) and c-Jun N-terminal kinase (JNK), as well as protein kinase B (AKT), are potential therapeutic targets for TNBC. Programmed death-ligand 1 (PD-L1) is implicated in TNBC progression and is associated with AKT and ERK signaling pathways. In addition, reactive oxygen species (ROS) act upstream of MAPK/AKT and PD-L1. In this study, we aimed to clarify the role of PD-L1 in TNBC progression and to delineate the underlying signaling mechanisms. **Methods:** Western blotting and reverse transcription–polymerase chain reaction were used to analyze protein and mRNA levels, respectively. Transwell migration and 3-[4,5-dimethylthiazol-2-yl]-2,5-diphenyltetrazolium bromide (MTT) assays were used to assess cell migration and proliferation, respectively. **Results:** The ERK inhibitor (PD98059) suppressed MDA-MB-231 cell migration but not proliferation, whereas PD-L1 siRNA and the ROS scavenger dithiothreitol (DTT) reduced both cell migration and proliferation. However, PD-L1 siRNA and DTT did not reduce the activities of ERK, JNK, or AKT. Whereas PD98059 and DTT suppressed PD-L1 protein expression, PD-L1 mRNA expression could be reduced by DTT only. Taken together, ROS and ERK may activate different pathways to regulate PD-L1 expression and MDA-MB-231 cell progression. Consistently, DTT combined with PD98059 additively inhibited MDA-MB-231 cell migration. Similar observations were noted in another TNBC cell line, Hs578T, which exhibits motility, but not in MDA-MB-453 cells, which lack motility. **Conclusion:** Since PD-L1 appears to function downstream of ERK and ROS and is required for TNBC progression, co-targeting both ERK and ROS signaling pathways may represent a promising therapeutic strategy for TNBC.

## 1 Introduction

Breast cancer (BC) is one of the most prevalent cancers worldwide and the leading cause of cancer-related death among women. BC is commonly classified into luminal A, luminal B, human epidermal growth factor receptor 2 (HER2)-positive, and triple-negative (TNBC) subtypes [1,2]. Among these, TNBC is the most aggressive, characterized by a poor prognosis and limited responsiveness to most targeted and immunotherapies [3].

Several oncogenic signaling pathways, including phosphoinositide 3-kinase (PI3K)/protein kinase B (AKT)/mTOR, vascular endothelial growth factor (VEGF), Janus kinase/signal transducer and activator of transcription 3 (JAK/STAT3), mitogen-activated protein kinases (MAPKs), epidermal growth factor receptor (EGFR), and Src, are involved in the progression of BC [4]. Among these, MAPK- and AKT-related signaling pathways are the most extensively studied in TNBC. MAPK and AKT are known to be overactivated in TNBC and are regarded as promising targets for inhibiting its progression [5]. For example, extracellular signal-regulated kinase (ERK) signaling is required for the progression of TNBC cells induced by CD147 and fibroblast growth factor 18 [6,7]. Moreover, PI3K/AKT/mTOR signaling can cross-talk with several oncogenic pathways to drive BC progression and contributes to drug resistance in BC management [8,9]. Specifically, the PI3K/AKT/mTOR pathway is essential for the survival and angiogenesis of TNBC [10]. However, compensatory signaling frequently occurs during MAPK- or AKT-targeted therapies, leading to resistance in TNBC treatment [11,12]. For example, the FDA-approved Mitogen-activated protein kinase kinase (MEK)1/2 dual inhibitors cobimetinib and trametinib, which suppress ERK activity, can induce activation of the B-RAF pathway [13]. Moreover, clinical trials involving FDA-approved inhibitors targeting PI3K (alpelisib) and mTOR (everolimus) have shown limited efficacy, largely due to drug-induced activation of alternative oncogenic pathways, including MAPK [14]. Drug-induced compensatory signaling in MAPK- and PI3K/AKT-targeted therapies has been attributed to cross-talk between these pathways [11,15,16]. One approach to address this issue is dual-targeted therapy for TNBC. For example, HPT-15, which simultaneously inhibits the mTOR and MAPK pathways, has been explored for the treatment of TNBC [17]. However, significant adverse effects have frequently been reported in clinical trials targeting both PI3K/AKT and MAPK pathways [18]. An alternative strategy is to identify upstream regulators of both MAPK and AKT signaling, which may represent more suitable therapeutic targets.

One potential upstream regulator of MAPK/AKT signaling in TNBC is programmed death-ligand 1 (PD-L1), a transmembrane protein belonging to the B7/CD28 family that regulates T-cell activation. PD-L1 is expressed not only in antigen-presenting cells but also in non-immune cells, including mesenchymal stem cells, epithelial cells, and tumor cells such as BC. The binding of PD-L1 to its receptor PD-1 activates the immune checkpoint axis, reducing the activity and survival of cytotoxic lymphocytes and thereby facilitating cancer progression. Indeed, PD-L1/PD-1 immune checkpoint–based immunotherapy has emerged as one of the most widely used approaches in contemporary cancer management [19]. In addition to its role as an immune checkpoint, PD-L1 also exerts tumor-intrinsic functions as a pro-tumorigenic factor, directly promoting cancer progression through the activation of proliferative and metastatic signaling pathways. Notably, Duaa et al. demonstrated that migration and invasion in the TNBC cell line MDA-MB-231 were reduced following PD-L1 knockout. This was accompanied by inhibition of AKT and ERK activity, along with decreased expression of downstream transcription factors such as c-Fos and c-Myc. Moreover, PD-L1–knockout MDA-MB-231 cells exhibited reduced tumor growth in the chick embryo chorioallantoic membrane model [20]. This suggests that PD-L1 acts as a positive regulator of MAPK/AKT signaling in TNBC progression and may serve as a more effective upstream therapeutic target. On the other hand, PD-L1 expression can be transcriptionally upregulated by various extracellular stimuli, including hypoxia, cytokines, and epidermal growth factor (EGF), mainly through the Ras/MAPK and/or PI3K/AKT pathways [20,21,22]. Taken together, these findings indicate that PD-L1 may engage in cross-talk with major oncogenic signaling pathways to promote TNBC progression.

Another potential upstream regulator of MAPK/AKT signaling is reactive oxygen species (ROS), including superoxide radicals (O_2_^−^), hydrogen peroxide (H_2_O_2_), and hydroxyl radicals (•OH). At low concentrations, ROS function as signaling modulators that promote cancer progression [23,24]. ROS can activate several oncogenic pathways, thereby initiating the progression of various cancers [25,26,27]. Specifically, ROS can activate MAPK and PI3K/AKT signaling to regulate the cell cycle and promote BC metastasis [28]. In contrast, at higher intracellular concentrations, ROS act as tumor suppressors by inducing senescence and cell death, thereby eliminating tumor cells [25]. Indeed, several studies have shown that ROS signaling mediates the tumor-suppressive effects of various anti-BC drugs [29,30]. To date, whether ROS serves as a common upstream activator of MAPK and PI3K/AKT in TNBC progression has not been fully established.

In this study, we aimed to clarify the cross-regulatory mechanisms among MAPK/AKT activation, ROS signaling, and PD-L1 expression in TNBC cell lines. In addition, combined targeting of these signaling molecules was investigated to determine whether it could more effectively suppress TNBC cell progression. These findings may provide more precise and effective strategies for the targeted therapy of TNBC.

## 2 Methods and Materials

### 2.1 Cell Cultures

MDA-MB-231 cells (HTB-26™, passage 480) were obtained from the American Type Culture Collection (Manassas, VA, USA) and confirmed to be free of mycoplasma contamination. Cells were cultured in Roswell Park Memorial Institute 1640 (RPMI-1640) medium (11875093, Thermo Fisher Scientific, Taiwan Co., Ltd., Taipei, Taiwan) supplemented with 10% heat-inactivated fetal bovine serum (AAFB0001-500, Biorion, New Taipei, Taiwan) and maintained at 37°C in a humidified atmosphere containing 5% CO_2_. Hs578T (BCRC 60120, passage 55) and MDA-MB-453 (BCRC 60429, passage 357) cells were obtained from the Bioresource Collection and Research Center (BCRC, Hsinchu, Taiwan) and confirmed to be free of mycoplasma contamination. Hs578T cells were cultured in Dulbecco’s Modified Eagle Medium (DMEM) (11965092, Thermo Fisher Scientific Taiwan Co., Ltd., Taipei, Taiwan) supplemented with 10% heat-inactivated FBS and 10 μg/mL bovine insulin (I0516, Sigma-Aldrich, St. Louis, MO, USA), and maintained at 37°C in a humidified atmosphere containing 5% CO_2_, whereas MDA-MB-453 cells were cultured in L-15 medium (11415064, Thermo Fisher Scientific Taiwan Co., Ltd., Taipei, Taiwan) supplemented with 10% heat-inactivated FBS (AAFB0001-500, Biorion) and maintained at 37°C under CO_2_-free conditions. All cell lines were authenticated by short tendem repeat (STR) profiling.

### 2.2 Antibodies and Chemicals

Rabbit polyclonal antibodies (Abs) against PD-L1 (1:1500, GTX104763), phosphorylated ERK (p-ERK; 1:2000, GTX635617), phosphorylated JNK (p-JNK; Thr183/Tyr185, 1:1000, GTX635799), phosphorylated AKT (p-AKT; Thr450, 1:2000, GTX641363), vimentin (1:1500, GTX100619), epithelial cell adhesion molecule (Ep-CAM) (1:2000, GTX636759), matrix metalloproteinase-2 (MMP2) (1:2000, GTX104577), and GAPDH (1:2000, GTX627408) were purchased from GeneTex (Irvine, CA, USA). Dithiothreitol (DTT; D0632), PD98059 (513000), SP600125 (S5567), and wortmannin (681675) were purchased from Sigma-Aldrich (St. Louis, MO, USA). DTT was dissolved in H_2_O to prepare a 5.0 M stock solution and used at final concentrations of 1.25–5.0 mM for cell treatment, whereas PD98059, SP600125, and wortmannin were dissolved in DMSO to prepare 20, 1.0 and 0.2 mM stocks solutions, respectively, used at final concentrations of 20–80, 0.2 and 0.04 μM for cell treatments.

### 2.3 RNA Interference

The expression of PD-L1 in MDA-MB-231 and Hs578T cells was transiently knocked down by transfection with PD-L1 siRNAs (75220, Santa Cruz Biotechnology Inc., Dallas, TX, USA) at a concentration of 12.5–25 nM using DharmaFECT 4 transfection reagent (T-2004-03, Horizon Discovery, Lafayette, CO, USA) according to the manufacturer’s protocol. Non-targeting siRNA (NT-siRNA; sc-37007, Santa Cruz Biotechnology Inc.), used at the same concentration as the PD-L1 siRNAs, contains scrambled sequences that do not target any known cellular mRNA and served as a negative control for transfection. Depletion of the indicated molecules was confirmed by Western blotting and reverse transcription–polymerase chain reaction (RT-PCR).

### 2.4 RT-PCR

RT-PCR was performed as described in a previous report [31] with modifications. Briefly, total RNA was extracted using TRIzol reagent (T9424, Sigma-Aldrich) according to the manufacturer’s instructions. Complementary DNA (cDNA) was synthesized from 1.0 μg of total RNA using the PrimeScript™ RT Reagent Kit (RR037A, Takara Bio Inc., Shiga, Japan) in a final reaction volume of 20 μL. Conventional PCR amplification was performed using PuriTaq DNA polymerase (PU-TQC-100, Purigo Biotech Inc., Taiwan) in a Veriti™ Thermal Cycler (Thermo Fisher Scientific, Waltham, MA, USA). One microliter of cDNA template was used for each reaction. PD-L1 PCR was conducted for 30 cycles, consisting of denaturation at 95°C for 25 s, annealing at 60°C for 30 s, and extension at 72°C for 25 s. GAPDH PCR was performed for 18 cycles, consisting of denaturation at 95°C for 15 s, annealing at 60°C for 30 s, and extension at 72°C for 27 s. A no-template negative control (nuclease-free water) was included to confirm the absence of contamination. The primer sequences used for RT-PCR were as follows: PD-L1 forward, 5′-ACCTCTGAACATGAACTGAC-3′; reverse, 5′-CTTTCATTTGGAGGATGTGC-3′; GAPDH forward, 5′-ACCACAGTCCATGCCATCAC-3′; reverse, 5′-TCCACCACCCTGTTGCTGTA-3′. The amplified PCR products were stained with Protech Clearview (PT-D2001, Protech Technology Enterprise Co., Ltd., Taipei, Taiwan), separated by electrophoresis on 2.0% agarose gels, and visualized using a UVP ChemStudio PLUS Bioimaging System (UVP, Upland, CA, USA). Band intensities were quantified using ImageJ software (version 1.50i; National Institutes of Health, Bethesda, MD, USA).

### 2.5 Western Blotting

Cell lysates (approximately 20–80 μg) were separated by polyacrylamide gel electrophoresis (PAGE) for 1.5 h, followed by electrotransfer to polyvinylidene fluoride (PVDF) membrane (Immobilon-P; IPVH00010, Merck Millipore Ltd., Cork, Ireland) at room temperature for 3.5 h. The membrane was then blocked with 5% skim milk for 1 h at room temperature, followed by incubation with the indicated rabbit polyclonal primary antibodies for 1 h at room temperature. Subsequently, the membranes were washed three times with Tris-buffered saline containing 0.1% Tween 20 (TBST) and incubated with goat anti-rabbit horseradish peroxidase (HRP)-conjugated secondary antibody (1:5000, GTX213110-01, GeneTex, Irvine, CA, USA) for 1 h at room temperature. Finally, the membranes were developed using HRP substrate (WBKLS0500, EMD Millipore, MA, USA) for 1–3 min, and protein bands were visualized using a UVP ChemStudio PLUS imaging system (Analytik Jena US, Upland, CA, USA). Band intensities were quantified using ImageJ software (version 1.50i; National Institutes of Health, Bethesda, MD, USA).

### 2.6 Proliferation Assay

Cell proliferation was assessed using a 3-[4,5-dimethylthiazol-2-yl]-2,5-diphenyltetrazolium bromide (MTT) assay (T0793, Biobasic, Amherst, NY, USA). MDA-MB-231 cells were seeded in 24-well plates (6 × 10^4^ cells/well) for overnight. After appropriate treatments for 48 h, cells were incubated with 0.5 mg/mL MTT in RPMI-1640 medium (11875093, Thermo Fisher Scientific) supplemented with 10% FBS (AAFB0001-500, Biorion) at 37°C for 2 h. The medium was then replaced with 0.5 mL DMSO to dissolve the formazan crystals for 5 min. Absorbance was measured at 595 nm using a Synergy HTX Multi-Mode Reader (Cold Spring Biotech Corp., New Taipei City, Taiwan).

### 2.7 Transwell Migration Assay

Cells were seeded into transwell migration inserts (PTEP24H48; pore size 8.0 μM; EMD Millipore, MA, USA) placed in 24-well plates at a density of 1.0 × 10^4^ cells/well. The following day, cells in the upper chamber were treated with the appropriate reagents for 48 h, while the lower chamber contained RPMI-1640 medium supplemented with 10% FBS only. After completion of the treatments, cells were fixed with 4% paraformaldehyde for 10 min and stained on both sides of the membrane with 0.3% crystal violet for 5 min. Non-migrated cells on the upper surface were removed using cotton swabs, and migrated cells on the lower surface were visualized under phase-contrast microscopy at 40× magnification. Quantification of migrated cells was performed by measuring the intensity of crystal violet staining across the entire lower membrane surface using ImageJ software (version 1.50i; National Institutes of Health, Bethesda, MD, USA).

### 2.8 Statistical Analysis

The Student’s *t*-test was used to determine statistically significant differences between the means of values obtained from Western blotting, cell migration, and proliferation analyses, with each indicated control group normalized to 1.0. Quantitative data are presented as mean ± standard deviation. A *p*-value of <0.05 (n > 3) was considered statistically significant.

## 3 Results

### 3.1 ERK Activity Was Specifically Required for the Migration of MDA-MB-231 Cells

**Figure 1:**
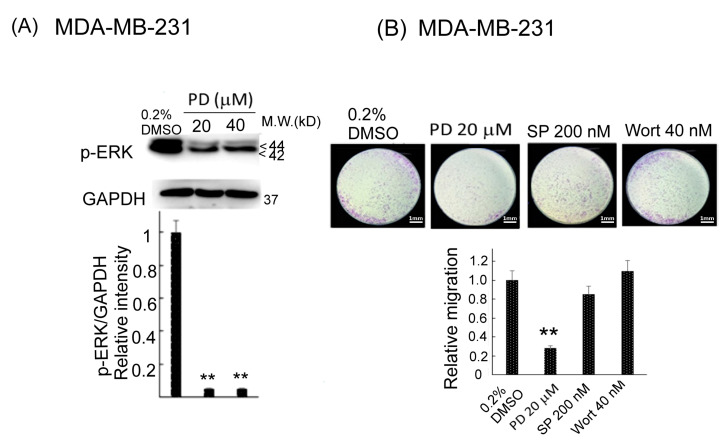
**Inhibition of extracellular signal-regulated kinase (ERK), but not JNK or AKT, suppresses MDA-MB-231 cell migration.** (**A**) MDA-MB-231 cells were treated with PD98059 (PD) for 48 h. Western blot analysis of p-ERK was performed, with GAPDH used as a loading control. The lower panel shows the quantitative data. Relative intensity was calculated by normalizing the 0.2% DMSO (vehicle) group to 1.0. (**B**) MDA-MB-231 cells were treated with PD, SP600125 (SP), or wortmannin (Wort) at the indicated concentrations for 48 h, followed by a transwell migration assay. The lower panel shows the quantitative data. Relative migration was calculated by normalizing the 0.2% DMSO (vehicle) group to 1.0. (**) represents the statistically significant difference (*p* < 0.01, N = 6, Student’s *t*-test) between the indicated samples and untreated (0.2% DMSO) group. Scale bar:1.0 mm.

First, we examined whether inhibitors of MAPKs, including ERK and c-Jun N-terminal kinase (JNK), as well as protein kinase B (AKT), effectively suppressed their respective target kinases by Western blot analysis of the corresponding active (phosphorylated) forms. As illustrated in Fig. 1A, ERK activity (p-ERK) was markedly reduced by 20 and 40 μM PD98059, an inhibitor of MEK (the upstream kinase of ERK), at concentrations approximately 10-fold higher than the IC50 of PD98059 in a cell-free system. However, AKT activity (p-AKT) was not reduced by its conventional inhibitor wortmannin (40 nM) (Supplementary Fig. S1), and JNK activity (p-JNK) was also not decreased by its conventional inhibitor SP600125 (200 nM) (data not shown), at concentrations approximately 10-fold higher than the IC50 values of both inhibitors in cell-free systems. The unexpected lack of efficacy of the AKT and JNK inhibitors in MDA-MB-231 cells may be due to compensatory signaling arising from pathway cross-talk, as described above. Consistently, PD98059 (20 μM) significantly reduced MDA-MB-231 cell migration by 75%, whereas SP600125 (200 nM) and wortmannin (40 nM) did not produce significant inhibition (Fig. 1B).

### 3.2 ERK Activity Was Required for PD-L1 Expression, Which Is Essential for MDA-MB-231 Cell Progression

**Figure 2:**
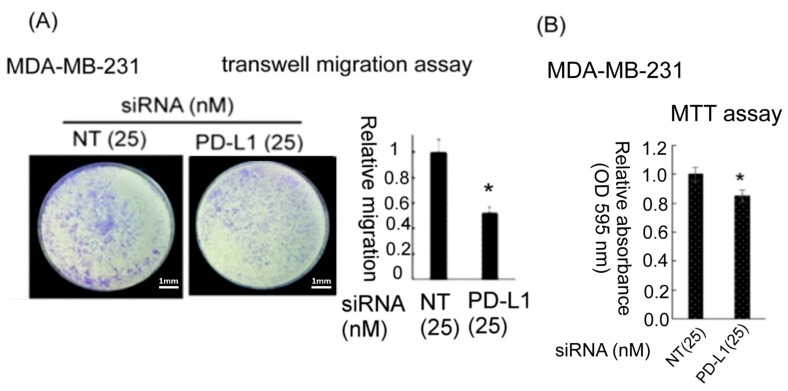
**Programmed death-ligand 1 (PD-L1) is required for MDA-MB-231 cell migration and proliferation.** (**A**) MDA-MB-231 cells were transfected with PD-L1 siRNA at the indicated concentrations for 48 h, followed by a transwell migration assay. Non-targeting siRNA (NT-siRNA) served as the control. Quantitative data are shown in the right panel. Relative migration was calculated by normalizing the NT-siRNA group to 1.0. (**B**) MDA-MB-231 cells were transfected with PD-L1 siRNA at the indicated concentrations for 48 h, followed by an MTT assay. NT-siRNA served as the control. Relative absorbance was calculated by normalizing the NT-siRNA group to 1.0. In (**A**) and (**B**), (*) represents the statistically significant difference (*p* < 0.05, N = 6, Student’s *t*-test) between the indicated samples and NT-siRNA group.

**Figure 3:**
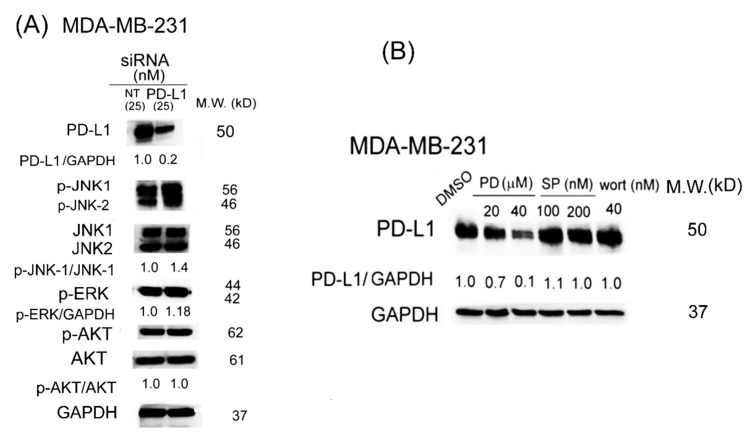
**ERK activity is required for PD-L1 protein expression, whereas PD-L1 is not required for activation of ERK, c-Jun N-terminal kinase (JNK), or protein kinase B (AKT) in MDA-MB-231 cells.** (**A**) MDA-MB-231 cells were transfected with PD-L1 siRNA or non-targeting (NT) siRNA for 48 h. Western blot analysis of the indicated molecules was performed. GAPDH and the corresponding total (unphosphorylated) kinases were used as internal controls. The values below each band represent the mean relative intensities from three independent experiments, normalized to GAPDH or the respective total kinase, with the NT-siRNA group set to 1.0. (**B**) MDA-MB-231 cells were treated with the indicated inhibitors for 48 h, followed by Western blot analysis of the indicated molecules. GAPDH was used as an internal control. The values below each band represent the mean relative intensities from three independent experiments, normalized to GAPDH, with the 0.2% DMSO group set to 1.0.

In a previous study, a PD-L1 gene knockout model was established using CRISPR-Cas9 lentiviral particles containing guide RNA (gRNA) targeting the PD-L1 genomic sequence to disrupt the PD-L1 gene in MDA-MB-231 cells [20]. The authors found that MAPK/AKT signaling, as well as cell proliferation, migration, and invasion, were reduced in PD-L1-knockout MDA-MB-231 cells, suggesting that PD-L1 is required for MAPK/AKT activation and MDA-MB-231 cell progression. Based on this evidence, we further investigated whether PD-L1 may act as an upstream regulator of MAPK/AKT by determining whether the reduction of PD-L1 expression produces similar effects. As illustrated in Fig. 2A,B, PD-L1 depletion using siRNA significantly suppressed MDA-MB-231 cell migration and proliferation by 50% and 18%, respectively. Surprisingly, depletion of PD-L1 did not reduce ERK or AKT activity and even slightly increased JNK activity (Fig. 3A). In contrast, PD98059 (20 and 40 μM), but not SP600125 (100–200 nM) or wortmannin (40 nM) suppressed PD-L1 expression in a dose-dependent manner by 30% and 90%, respectively (Fig. 3B).

### 3.3 Involvement of ROS in Regulating PD-L1 Expression during MDA-MB-231 Cell Progression

**Figure 4:**
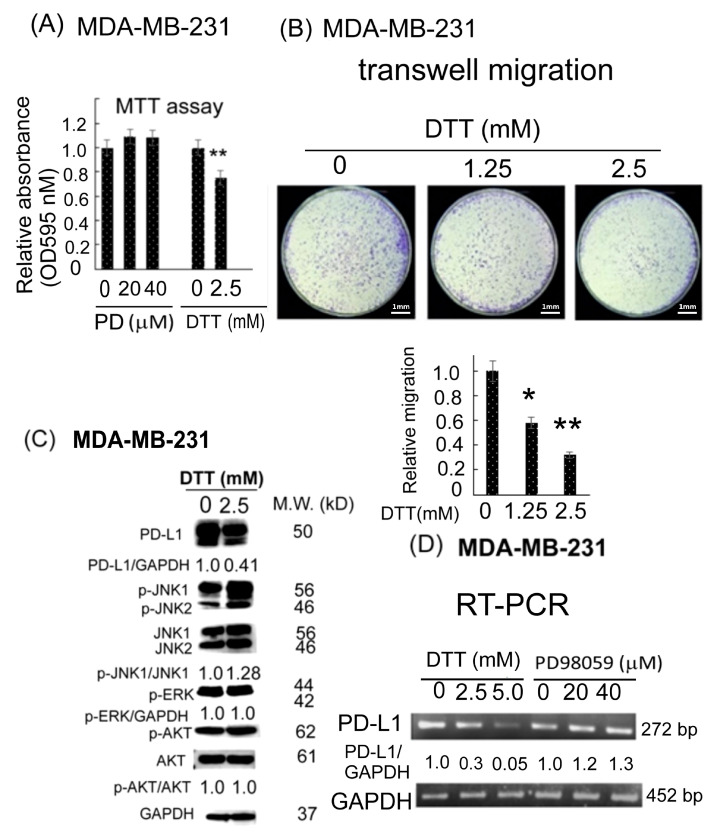
**ROS are required for cell proliferation, migration, and PD-L1 expression in MDA-MB-231 cells.** (**A**) MDA-MB-231 cells were treated with DTT or PD98059 (PD) at the indicated concentrations for 48 h, followed by a MTT assay. Relative absorbance was calculated by normalizing the untreated (zero) group to 1.0. (**B**) MDA-MB-231 cells were treated with DTT at the indicated concentrations for 48 h, followed by a transwell migration assay. In lower panel, relative migration was quantified by normalizing the untreated (zero) group to 1.0. (**C**) MDA-MB-231 cells were treated with DTT at the indicated concentrations for 48 h, followed by Western blot analysis of the indicated molecules. GAPDH and the corresponding total (unphosphorylated) kinases were used as internal controls. The values below each band represent the mean relative intensities from averaged data, normalized to GAPDH or the respective total kinase, with the untreated (zero) group set to 1.0. (**D**) MDA-MB-231 cells were treated with DTT or PD98059 at the indicated concentrations for 48 h, followed by RT-PCR analysis of PD-L1. GAPDH was used as an internal control. The values represent the mean relative PD-L1/GAPDH intensities, normalized to the untreated (zero) group set to 1.0. In (**A**) and (**B**), (*, **) represent the statistically significant difference [*p* < 0.05, *p* < 0.01, N = 6 (for A) or 3 (for B), Student’s *t*-test] between the indicated samples and untreated (DTT 0) group.

Since PD-L1 depletion did not affect ERK activation (Fig. 3A) while ERK inhibition suppressed PD-L1 expression (Fig. 3B), these findings suggest that PD-L1 functions downstream of ERK. Therefore, other upstream pathways may regulate MAPK/AKT activity and/or PD-L1 expression during MDA-MB-231 cell progression. One potential candidate is ROS-related signaling, which is known to be involved in MAPK/AKT pathways in many cancers, including BC [28]. To investigate this, dithiothreitol (DTT), a ROS scavenger, was used. As illustrated in Fig. 4A, 2.5 mM DTT, but not 20 or 40 μM PD98059, significantly reduced MDA-MB-231 cell proliferation by 23%. Moreover, DTT at 1.25 and 2.5 mM reduced MDA-MB-231 cell migration by 42% and 65%, respectively, in a dose-dependent manner (Fig. 4B). However, 2.5 mM DTT did not reduce ERK or AKT activity and even slightly increased JNK activity (phosphorylated JNK, p-JNK) (Fig. 4C). Collectively, these findings were consistent with those observed in PD-L1 knockdown cells described above (see comparison of Fig. 4 with Fig. 2A,B and Fig. 3A). Importantly, 2.5 mM DTT markedly reduced PD-L1 protein expression by 59% (Fig. 4C). In addition, PD-L1 mRNA levels were decreased by 2.5 and 5.0 mM DTT in a dose-dependent manner by 70% and 95%, respectively (Fig. 4D). In contrast, no reduction in PD-L1 mRNA was observed in PD98059-treated cells (Fig. 4D). Taken together, these results suggest that ROS, rather than ERK, act as a major positive regulator of PD-L1 transcription and expression, thereby contributing to MDA-MB-231 cell progression. To validate the role of ROS in regulating PD-L1 expression and oncogenic signaling in MDA-MB-231 cell migration, catalase (CAT), another ROS scavenger known to be a hydrogen peroxide (H_2_O_2_)-degrading enzyme, was employed. Similar to the effects of DTT, CAT (1 × 10^4^, 2 × 10^4^, and 3 × 1^4^ U/mL) decreased cell migration by 32%, 50%, and 70%, respectively, in a dose-dependent manner (Supplementary Fig. S2). At the molecular level, CAT (1 × 10^4^ U/mL) also suppressed PD-L1 expression by 62% but did not affect ERK or AKT activity (Supplementary Fig. S3). Overall, the effects of CAT on PD-L1 expression, MAPK/AKT signaling, and MDA-MB-231 cell migration were consistent with those of DTT. However, CAT significantly inhibited JNK activity by 80% (Supplementary Fig. S3), in contrast to DTT, which slightly increased JNK activity by 28% (Fig. 4C). The underlying mechanisms of this discrepancy are discussed in Section 4.3.

### 3.4 ERK Signaling and ROS-Regulated PD-L1 Pathways Mediate Distinct Mesenchymal Gene Expression

**Figure 5:**
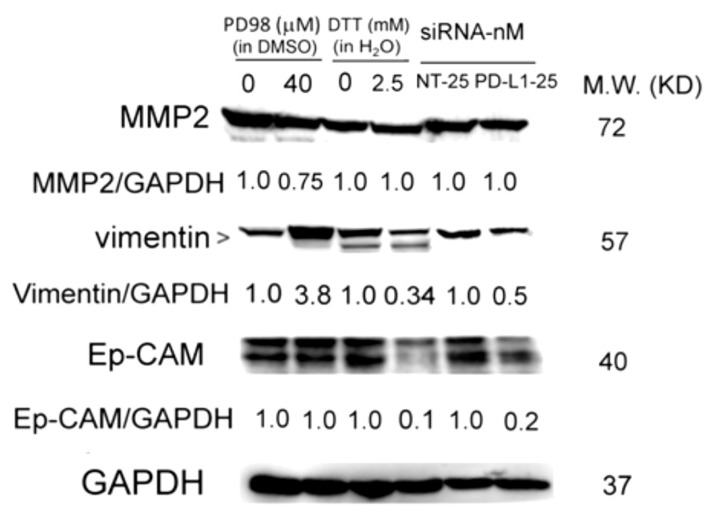
**ERK and ROS/PD-L1 pathways mediate differential expression of mesenchymal genes.** MDA-MB-231 cells were treated with PD98059 (PD98) or DTT, or transfected with PD-L1 siRNA at the indicated concentrations for 48 h. Western blot analysis of the indicated mesenchymal markers was performed. GAPDH was used as an internal control. The values below each band represent the mean relative intensities, normalized to GAPDH, from three independent experiments (n = 3). Data were normalized to the DMSO, H_2_O, and NT-siRNA groups, which were set to 1.0 for PD98059, DTT, and PD-L1 siRNA treatments, respectively.

Thus far, ERK and ROS appear to act upstream of PD-L1 (at the level of protein expression) and are both required for cell migration; however, ERK activity was not affected by the ROS scavenger DTT (Fig. 4C). Moreover, ROS, but not ERK activity, was required for MDA-MB-231 cell proliferation (Fig. 4A) and for PD-L1 transcription (mRNA level) (Fig. 4D). Collectively, these results suggest that ERK and ROS operate through distinct signaling pathways. This can be further supported by examining whether known mesenchymal genes in TNBC, including vimentin, Ep-CAM, and MMP2, are differentially regulated by ERK and ROS. As shown by Western blot analysis in Fig. 5, Ep-CAM expression was reduced by 2.5 mM DTT and 25 nM PD-L1 siRNA by 90% and 80%, respectively, compared with H_2_O and NT-siRNA controls. Similarly, vimentin expression was reduced by 66% and 50%, respectively, under the same treatments in MDA-MB-231 cells. In contrast, Ep-CAM expression was not reduced by 40 μM PD98059, while vimentin expression was markedly increased by PD98059 by 3.8-fold compared with DMSO. On the other hand, MMP2 expression was significantly reduced by PD98059 (by 25%) but was not affected by DTT or PD-L1 siRNA. Consistent with the effects of DTT, the other ROS scavenger, CAT, also reduced the mesenchymal markers Ep-CAM and vimentin by 52% and 48%, respectively, but did not affect MMP2 expression (Supplementary Fig. S3). Taken together, these results suggest that ERK signaling and the ROS-regulated PD-L1 axis mediate distinct molecular pathways controlling different mesenchymal markers involved in MDA-MB-231 cell progression.

### 3.5 Dual Blockade of ROS and ERK Signaling Suppresses MDA-MB-231 Cell Migration More Effectively than Single Treatment

**Figure 6:**
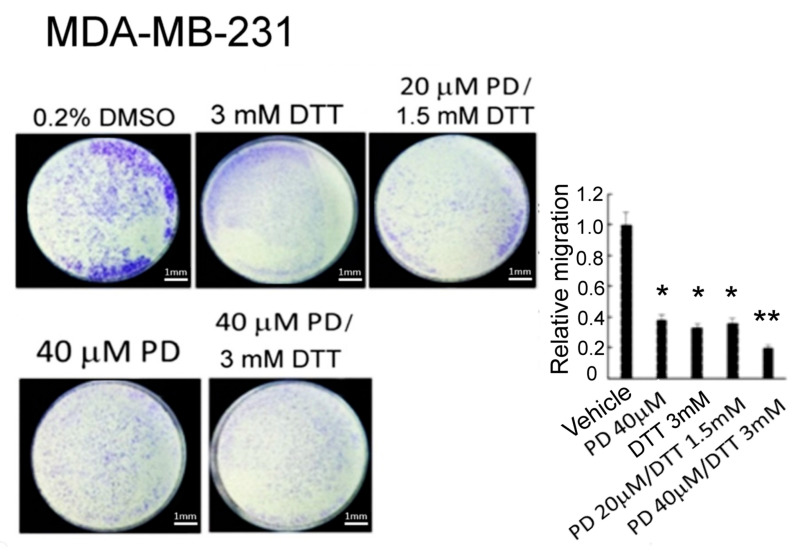
**Dual blockade of ROS and ERK signaling suppresses MDA-MB-231 cell migration more effectively than single treatment.** MDA-MB-231 cells were treated with PD98059 (PD), DTT, or their combination at the indicated concentrations, followed by a transwell migration assay. In the right panel, relative migration was calculated by normalizing the vehicle group (0.2% DMSO) to 1.0. (*, **) represent the statistically significant difference (*p* < 0.05, *p* < 0.01, N = 3, Student’s *t*-test) between the indicated samples and untreated (Vehicle) group.

Since ERK and ROS operate in distinct pathways, as described above, co-targeting both signaling molecules may offer improved therapeutic efficacy. To evaluate this, MDA-MB-231 cells were treated with DTT and PD98059, either alone or in combination. As illustrated in Fig. 6, combined treatment with 40 μM PD98059 and 3 mM DTT suppressed MDA-MB-231 cell migration by 80%, which was greater than the effects of 40 μM PD98059 (61%) or 3 mM DTT (65%) alone. On the other hand, combining both agents at half doses (20 μM PD98059 plus 1.5 mM DTT) produced a similar inhibitory effect (61%) to that observed with either higher-dose single treatment (40 μM PD98059 or 3 mM DTT).

### 3.6 Hs578T TNBC Cells Exhibit Similar Molecular and Cellular Characteristics to MDA-MB-231 Cells

To validate the molecular pathway of cell migration identified in MDA-MB-231 cells, two additional TNBC cell lines, Hs578T and MDA-MB-453, were examined. Hs578T cells exhibited strong spreading on culture dishes similar to MDA-MB-231 cells but showed relatively slower growth rates (data not shown), whereas MDA-MB-453 cells proliferated faster than Hs578T cells but lacked spreading ability, with more than 90% of cells displaying a rounded morphology (data not shown). In the transwell migration assay, Hs578T cell motility was reduced by DTT (4.0 mM), PD-L1 siRNA (25 nM), and PD98059 (80 μM) by 78%, 58%, and 43%, respectively, after 48 h (Supplementary Fig. S4A). In contrast, the basal motility of untreated MDA-MB-453 cells was minimal; therefore, the effects of PD98059 (80 μM) and DTT (2.5 mM) on these cells were not significant (Supplementary Fig. S4B). At the molecular level, PD-L1 expression was abundant in Hs578T cells and was significantly reduced by PD98059 (by 87%) (Supplementary Fig. S5A), whereas PD-L1 expression was very low in MDA-MB-453 cells, and its suppression by PD98059 and DTT was not significant (data not shown). In contrast, p-ERK and p-AKT levels were not significantly affected by PD-L1 siRNA in Hs578T cells (Supplementary Fig. S5B). The knockdown efficiency of 25 nM PD-L1 siRNA was approximately 49% (Supplementary Fig. S5B). Moreover, DTT (1.25–2.5 mM) reduced PD-L1 expression in a dose-dependent manner (by 15–42%) (Supplementary Fig. S5C), whereas p-ERK levels were not significantly affected by 1.25 and 2.5 mM DTT (Supplementary Fig. S5C). Overall, these findings were consistent with those observed in MDA-MB-231 cells (see comparison of Supplementary Fig. S5 with Fig. 2, Fig. 3 and Fig. 4). One exception is that p-AKT was increased by 1.25 and 2.5 mM DTT (by 50–80%) in Hs578T cells (Supplementary Fig. S5C), which is inconsistent with the lack of effect observed in MDA-MB-231 cells (Fig. 3A). Collectively, ERK- and ROS-regulated PD-L1 expression appears to be crucial for the migration of Hs578T cells but not for the non-motile MDA-MB-453 cells. Regarding mesenchymal markers regulated by ROS, ERK, and PD-L1 in Hs578T cells, Ep-CAM expression was markedly reduced by PD-L1 siRNA (by 90%) (Supplementary Fig. S5B), whereas vimentin was suppressed by DTT (1.25 and 2.5 mM) in a dose-dependent manner (by 88–92%) (Supplementary Fig. S5C) and was upregulated by PD98059 (by 70%) (Supplementary Fig. S5A). In contrast, MMP2 expression was not significantly affected by PD-L1 siRNA (Supplementary Fig. S5B) or DTT (data not shown). In general, these results were consistent with those observed in MDA-MB-231 cells (Fig. 5). However, in Hs578T cells, vimentin was upregulated by PD-L1 siRNA (Supplementary Fig. S5B), and Ep-CAM was not affected by DTT (data not shown). In addition, MMP2 was not suppressed by PD98059 (Supplementary Fig. S5A). These findings differ from those observed in MDA-MB-231 cells (Fig. 5), suggesting that the regulatory mechanisms of mesenchymal markers by ROS, ERK, and PD-L1 may vary slightly between MDA-MB-231 and Hs578T cells. Overall, the molecular and cellular effects of ERK inhibition, ROS scavenging, and PD-L1 knockdown in Hs578T cells showed approximately 80–90% similarity to those observed in MDA-MB-231 cells.

## 4 Discussion

### 4.1 Activity of ERK, but Not JNK or AKT, Is Required for MDA-MB-231 Cell Migration

Previously, MAPK, including ERK and JNK, as well as AKT, were known to be involved in the progression of TNBC [6,7,8,9,10]. In this study, we demonstrated that the ERK inhibitor PD98059, but not the AKT inhibitor wortmannin or the JNK inhibitor SP600125, effectively suppressed migration of the TNBC cell line MDA-MB-231 (Fig. 1B). The inability of wortmannin and SP600125 to inhibit MDA-MB-231 migration was associated with their lack of efficacy in suppressing their respective target kinases in these cells (Supplementary Fig. S1 and data not shown); however, the underlying mechanisms require further investigation. It has been reported that the complex cellular functions of AKT and the activation of compensatory feedback signaling limit the efficacy of single-target inhibition of the PI3K–AKT pathway [14,32,33]. On the other hand, although JNK has been implicated in TNBC progression and is considered a potential therapeutic target, the context-dependent roles of JNK isoforms (JNK1, JNK2, and JNK3) and their complex cross-talk with other signaling pathways often limit the effectiveness of JNK inhibition [34,35]. Therefore, the AKT and JNK inhibitors used in this study (wortmannin and SP600125, respectively) may not specifically or effectively suppress their target kinases and may even induce compensatory signaling in MDA-MB-231 cells. Future studies should focus on developing more specific and potent AKT and JNK inhibitors to improve therapeutic strategies for TNBC.

### 4.2 ERK and ROS Signaling Regulate PD-L1 Expression through Distinct Mechanisms in MDA-MB-231 Cell Migration

At the outset of this study on the role of PD-L1 in regulating MDA-MB-231 cell progression, we anticipated that PD-L1 might positively regulate MAPK and AKT activity based on previous reports. For example, reduced migration and invasion, accompanied by decreased AKT and ERK activities, were observed in PD-L1-knockout MDA-MB-231 cells generated using CRISPR-Cas9 lentiviral particle technology [20]. However, we found that PD-L1 depletion did not reduce the activity of MAPK kinases (including ERK and JNK) or AKT (Fig. 3A), suggesting that PD-L1 does not act as an upstream regulator of these signaling kinases. The discrepancy between our findings and those of the aforementioned study may be attributed to differences in cellular context arising from distinct experimental systems. The observed downregulation of AKT and ERK activities in PD-L1-knockout MDA-MB-231 cells may result from long-term and complex molecular adaptations following PD-L1 gene ablation and sustained loss of PD-L1 protein, which could alter overall signal transduction status. In contrast, in our study, PD-L1 knockdown produced only a transient reduction in PD-L1 protein, and the associated molecular effects are likely more immediate and direct. These findings suggest that PD-L1 protein itself is not required for MAPK/AKT signaling. Notably, a previous study reported that PD-L1 depletion by siRNA reduced MDA-MB-231 cell proliferation and migration and induced apoptosis; however, the effects on associated oncogenic signaling pathways and downstream transcriptional or translational mechanisms were not investigated [36]. In contrast, our results demonstrated that PD-L1 protein expression was reduced by the ERK inhibitor PD98059 (Fig. 3B). Indeed, numerous studies have highlighted a complex relationship between PD-L1 and epithelial–mesenchymal transition (EMT) signaling, in which AKT and ERK often act upstream of PD-L1 in various contexts [37]. For example, EMT-related transcription factors, including the Zinc finger E-box binding homeobox (ZEB), Twist, and Snail families, have been shown to regulate PD-L1 expression through the MAPK pathway in TNBC cells. However, in our study, PD-L1 mRNA levels were not altered by PD98059 in MDA-MB-231 cells (Fig. 4D), suggesting that PD-L1 protein may also be regulated by the ERK pathway at post-transcriptional or post-translational levels in TNBC.

On the other hand, we found that ROS signaling is required for PD-L1 expression at both the mRNA and protein levels. Moreover, the ROS scavenger DTT suppressed MDA-MB-231 cell migration and proliferation, accompanied by decreased expression of the same mesenchymal markers (vimentin and Ep-CAM) observed in PD-L1 knockdown cells (Fig. 5). Collectively, these results suggest that ROS acts as a crucial upstream regulator of PD-L1. This is consistent with previous review reports indicating that ROS promotes or is required for PD-L1 expression in several cancers [38]. Taken together, a ROS-triggered PD-L1 axis can be proposed, mediating the expression of mesenchymal markers and thereby promoting MDA-MB-231 cell migration and proliferation.

### 4.3 Validation of the Role of ROS in Regulating PD-L1 Expression in MDA-MB-231 Cell Progression

We investigated the role of ROS in regulating PD-L1 expression and MDA-MB-231 cell migration using two ROS scavengers, CAT and DTT. Similar to the effects of DTT, CAT reduced cell migration (compare Supplementary Fig. S2 with Fig. 4B) and inhibited PD-L1 expression without affecting ERK or AKT activity (compare Supplementary Fig. S3 with Fig. 4C). CAT also decreased the expression of the mesenchymal markers Ep-CAM and vimentin, but not MMP2 (Supplementary Fig. S3), consistent with the effects observed for DTT (Fig. 5). Taken together, the molecular and cellular effects of these two ROS scavengers in MDA-MB-231 cells were highly similar, further supporting the role of ROS as a key regulator of PD-L1 expression in TNBC progression. One exception is the results regarding JNK. While DTT did not reduce JNK activity and even slightly increased it (Fig. 4C), CAT significantly decreased JNK activity (Supplementary Fig. S3). This discrepancy may be attributed to differences in how these agents modulate ROS signaling, as DTT is a general antioxidant whereas CAT specifically degrades H_2_O_2_. H_2_O_2_ is widely recognized as a highly diffusible and key ROS species involved in regulating signal transduction [39]. Specifically, H_2_O_2_ is known to be important for the activation of MAPKs, including JNK [25]. Thus, compared with DTT, CAT may exert a stronger scavenging effect on H_2_O_2_, leading to a greater impact on signaling kinases such as JNK. This suggests that JNK may also lie downstream of H_2_O_2_. However, inhibition of JNK did not affect PD-L1 protein expression in MDA-MB-231 cells (Fig. 3B). Therefore, H_2_O_2_-regulated JNK activation may be involved in other oncogenic pathways unrelated to PD-L1, which warrants further investigation.

### 4.4 The Possible Link between ROS and ERK in TNBC

As described in the Introduction, previous studies have suggested that ROS functions as an upstream regulator of MAPK/AKT signaling in tumor progression. However, in our study, we did not observe a positive regulatory effect of ROS on MAPK/AKT in TNBC, as ROS scavenging did not reduce the activities of ERK, JNK, or AKT in either MDA-MB-231 (Fig. 4C and Supplementary Fig. S3) or Hs578T cells (Supplementary Fig. S5C). In tumor cells, the relationship between ROS and MAPK signaling is complex. On one hand, ROS can enhance MAPK signaling [40], contributing to uncontrolled proliferation and therapy resistance. On the other hand, oncogenic MAPK signaling can promote mitochondrial ROS production, thereby further sustaining ERK activation [41].

Thus, ROS and MAPK may function as both upstream and downstream regulators of each other within the signaling network. Notably, a recent study demonstrated that antioxidant treatment did not decrease, but instead increased ERK phosphorylation in MDA-MB-231 cells. Conversely, treatment with the potent MEK inhibitor PD0325901 reduced ROS levels in MDA-MB-231 cells [42]. Since we observed that the ROS scavenger DTT did not decrease—and even slightly increased—the activities of MAPKs (including ERK and JNK) and AKT in both MDA-MB-231 and Hs578T cells (Fig. 4C and Supplementary Fig. S5C), whether ERK acts upstream of ROS in TNBC remains an interesting question that warrants further investigation.

### 4.5 Multiple Pathways Mediate TNBC Progression: Implications for Combined Targeting Strategies

**Figure 7:**
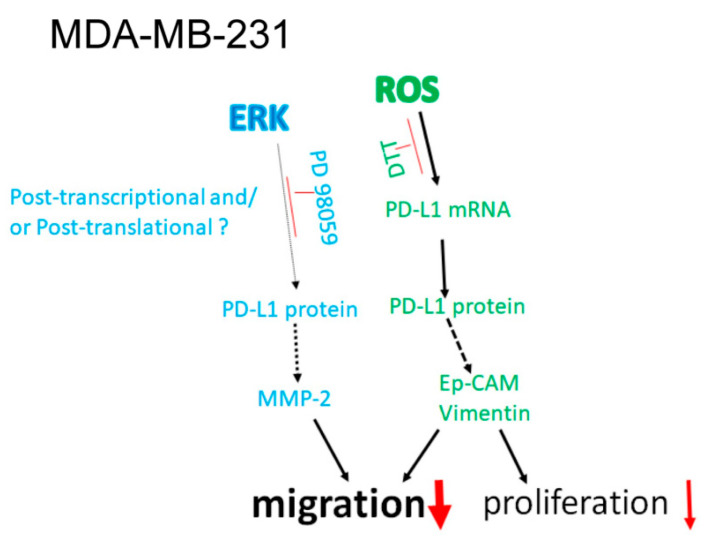
**PD-L1-mediated ERK and ROS signaling pathways as promising targets for preventing MDA-MB-231 cell progression.** ERK and ROS regulate PD-L1 expression via different mechanisms for regulating diverse mesenchymal genes, leading to migration (under both ERK and ROS pathways) and proliferation (under ROS pathway) in MDA-MB-231 cells. Co-targeting ERK and ROS with indicated antagonists resulted in greater suppression of MDA-MB-231 cell migration, whereas single targeting ROS significantly decreased cell proliferation. Solid and dashed arrow lines indicate “defined” and “undefined” regulatory events, respectively. Small red lines ⊥ indicate the inhibitory effect exerted by the indicated inhibitors. The red arrow **↓** represents the downregulation of the indicated phenotypical changes in MDA-MB-231 cells.

In previous TNBC clinical trials, the benefit of single-agent conventional anticancer therapy or immunotherapy has been limited due to tumor heterogeneity and drug resistance. As a result, various combination therapies targeting both the immune system (e.g., PD-L1) and oncogenic receptors (such as epidermal growth facor receptor and vascular endothelial growth factor receptor), as well as intracellular signaling kinases including MAPK and Src, have been developed [43]. In this study, we found that ROS and ERK signaling operate within distinct pathways to regulate different patterns of PD-L1 expression (Fig. 4C) and diverse mesenchymal marker expression (Fig. 5). These pathways converge to produce similar effects on cell migration (Fig. 1B, Fig. 4B) but different effects on cell proliferation (Fig. 4A) in MDA-MB-231 cells, suggesting that a combined targeting strategy may achieve higher therapeutic efficacy. This issue was partially addressed by the finding that combined targeting of ROS and ERK more effectively suppressed MDA-MB-231 cell migration than either single treatment alone (Fig. 6). The proposed mechanism is summarized in the schematic model (Fig. 7).

Although we have delineated a pathway linking PD-L1 expression to the migration of MDA-MB-231 and Hs578T cells, and a combined ROS- and ERK-based targeting strategy appears promising, it remains unclear whether the ROS and ERK inhibitors used in this study are equally effective in other TNBC cell lines, such as MDA-MB-468 and BT-549, or in patient-derived TNBC cells. This warrants further investigation to establish a generalizable molecular mechanism underlying tumor-intrinsic PD-L1 oncogenic activity in TNBC.

In conclusion, since PD-L1 functions not only as an immune checkpoint molecule but also as a pro-tumorigenic factor, we propose that co-targeting the ERK- and ROS-regulated PD-L1 axis within tumor cells, together with PD-L1-based immunotherapy in the tumor microenvironment, may represent a more promising therapeutic strategy for future preclinical and clinical studies in TNBC.

## Data Availability

All data generated or analyzed during this study are included in this published article and its supplementary data.

## Supplementary Materials

The supplementary material is available online at https://www.techscience.com/doi/10.32604/or.2026.077693/s1.

## Abbreviations

triple-negative breast cancer	TNBC
mitogen-activated protein kinases	MAPK
programmed death-ligand 1	PD-L1
reactive oxygen species	ROS
epithelial cell adhesion molecule	Ep-CAM
metalloproteinase-2	MMP2
dithiothreitol	DTT
