# Supplementary material for: Extracellular Signal-Regulated Kinase and Reactive Oxygen Species Regulate PD-L1 to Promote Migration and Proliferation of Triple-Negative Breast Cancer MDA-MB-231 Cells
Source: Oncol Res. 2026 Jul 16;34(8):17. doi: 10.32604/or.2026.077693 (PMC13397364; doi:10.32604/or.2026.077693)
Supplement: Supplementary file 1 [file OncolRes-34-77693-s001.zip › TSP_OR_77693-s001.docx]

**Supplementary Figures**


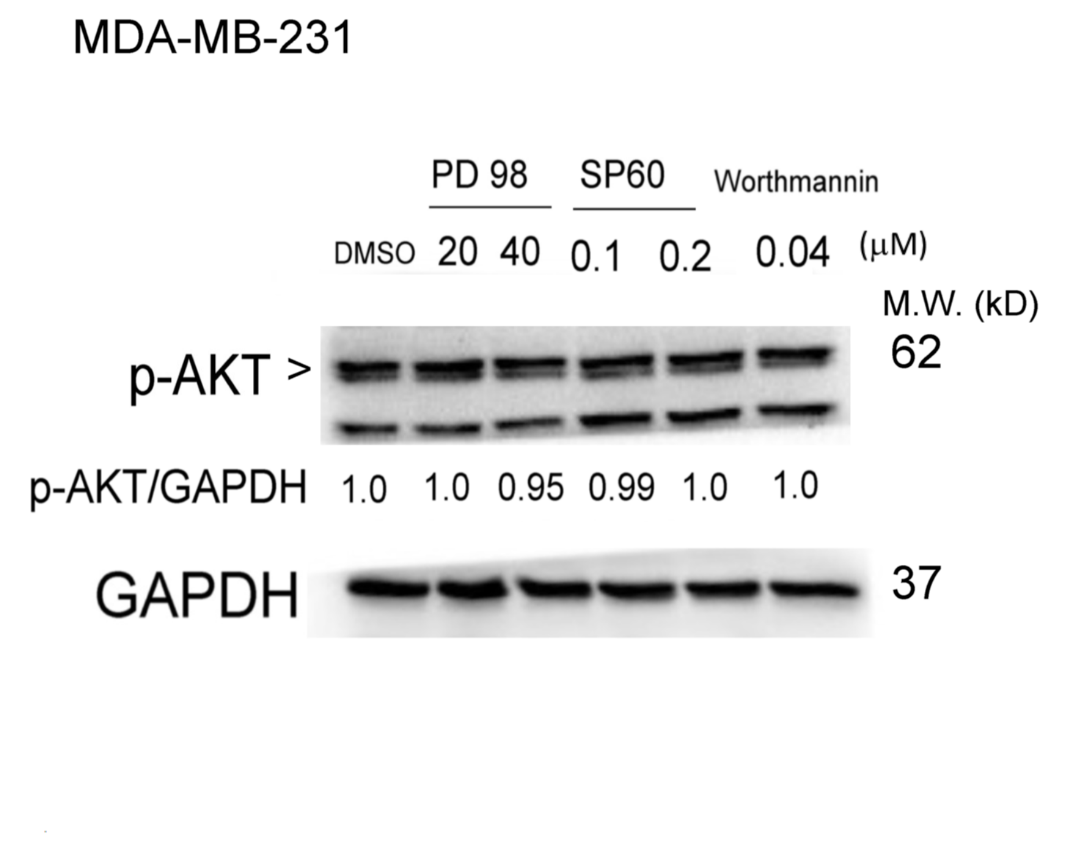


**Supplementary Fig.1 Worthmannin could not suppress AKT activity in MDA-MB-231 cells**

MDA-MB-231 cells were treated with the indicated inhibitors at indicated concentration for 48h. Western blot of p-AKT was performed. GAPDH was used as an internal control. The numbers below each band represent the relative intensities of the averages of three reproducible data of p-AKT *vs* that of GAPDH, taking the untreated (DMSO) group as 1.0. PD 98: PD98059; SP60:SP600125.


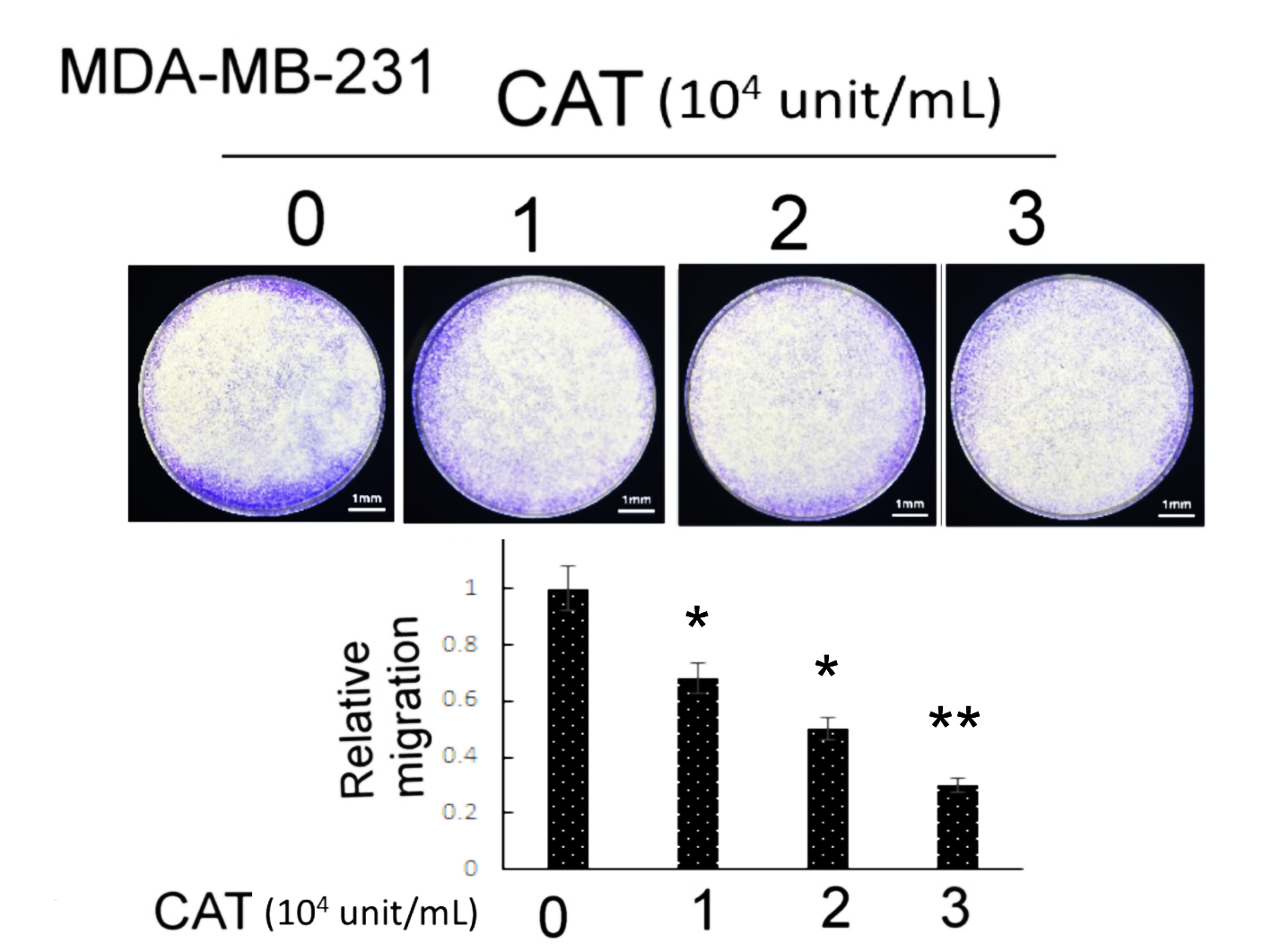


**Supplementary Fig.2 Catalase suppressed MDA-MB-231 cell migration in a dose-dependent manner.** MDA-MB-231 cells were treated with the ROS scavenger catalase (CAT) at the indicated concentration for 48h. The trans-well migration assay was performed. Quantitative data is shown in the low panel. Relative migration was calculated by taking the untreated (CAT 0) group as 1.0. The data shown were the average of three reproducible results. (*, **) represent the statistically significant difference (P < 0.05, P < 0.01, N = 3, Student’s t-test) between the indicated samples and untreated (CAT 0) group.


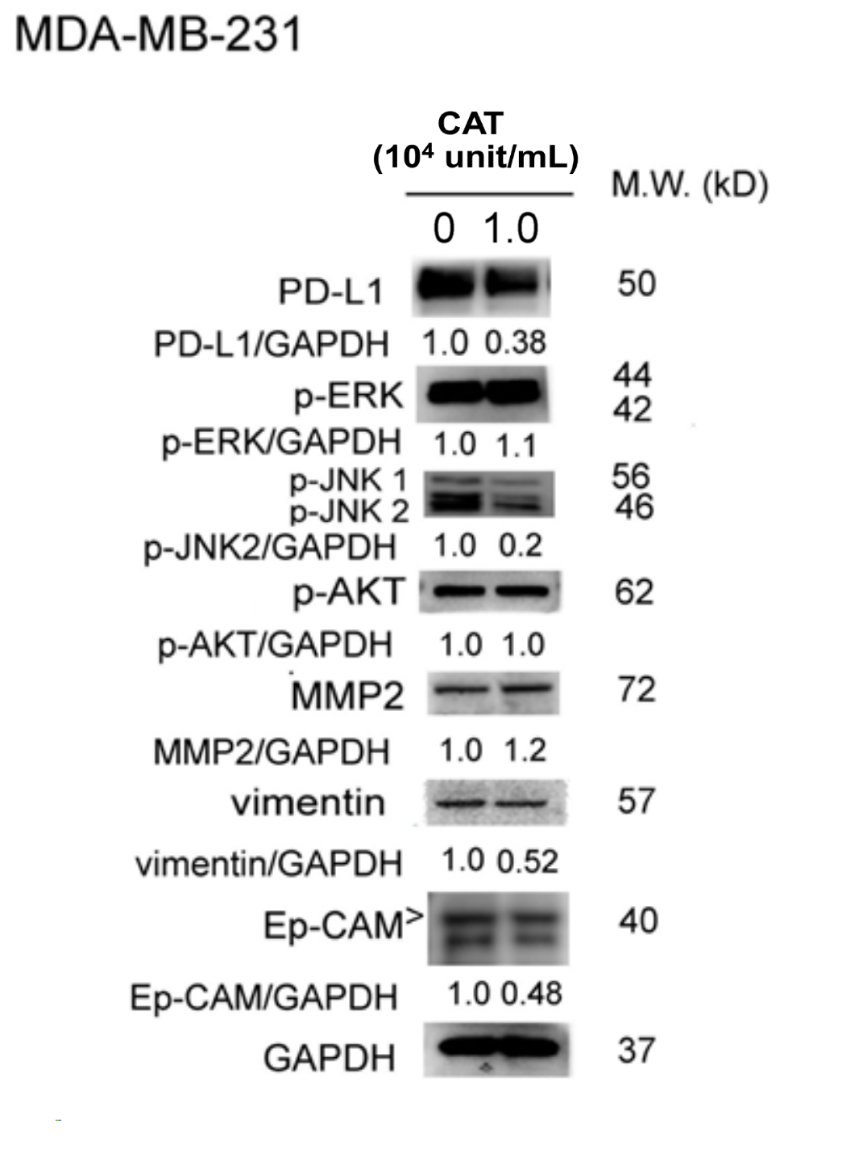


**Supplementary Fig. 3 Catalase suppressed PD-L1 expression and regulated oncogenic signaling and mesenchymal markers in MDA-MB-231.** MDA-MB-231 cells were treated with the ROS scavenger catalase (CAT) at the indicated concentration for 48h. Western blot of the indicated molecules was performed. GAPDH was used as an internal control. The numbers below each band represent the relative intensities of the averages of three reproducible data of indicated molecules *vs* that of GAPDH, taking the untreated (CAT 0) group as 1.0.

**
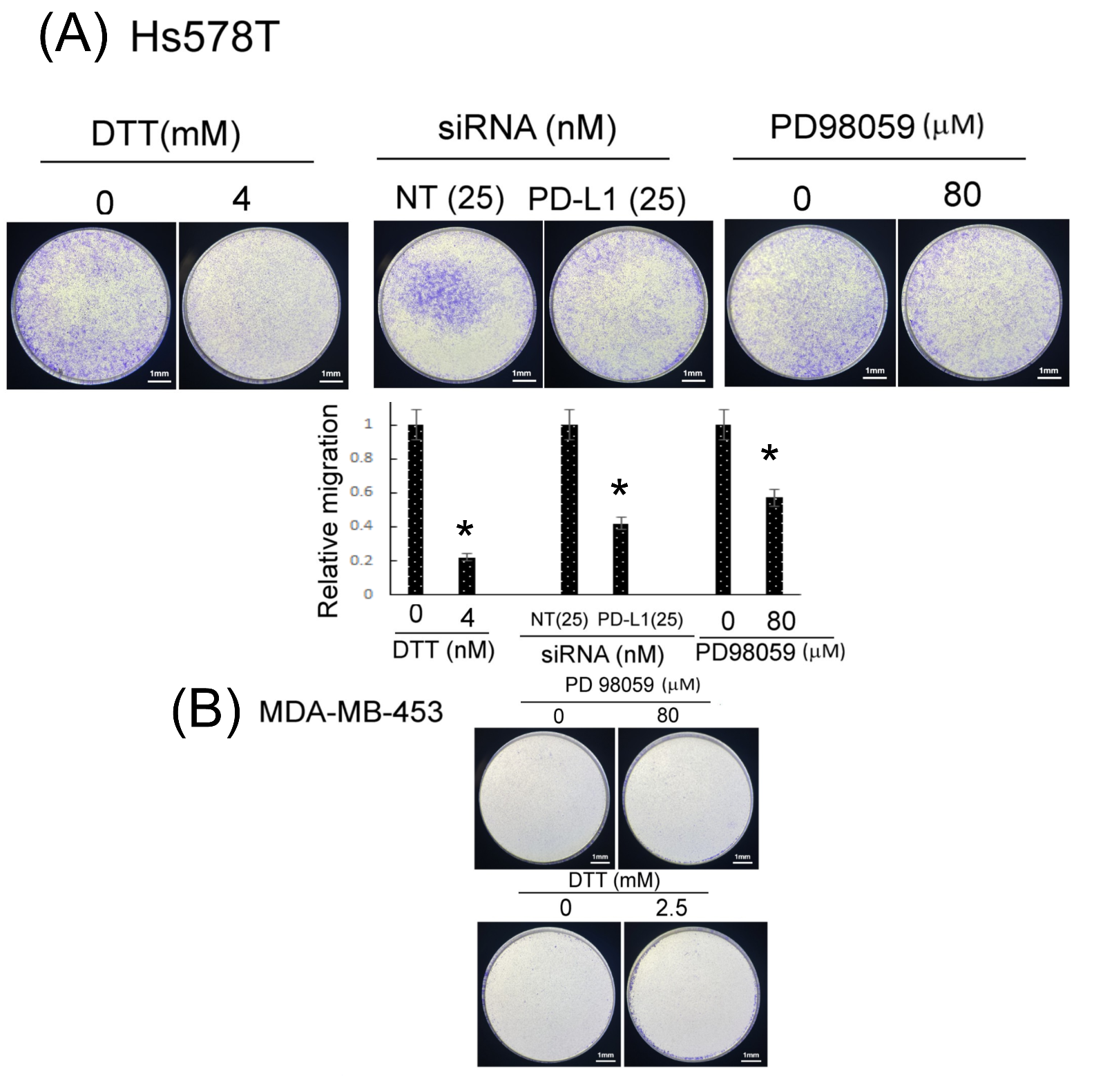
**

**Supplementary Fig.4 ROS scavenger, ERK inhibitor and PD-L1 knockdown inhibit cell migration of Hs578T but not MDA-MB-453 cells.**

(A) Hs578T cells were treated with the ROS scavenger DTT (left panel), transfected with PD-L1-siRNA (middle panel) or PD98059 (right panel), at the indicated concentration for 48h; trans-well migration assay was performed. Quantitative data for each treatment are shown in the low panel. Relative migration was calculated by taking H_2_O (for DTT), NT-siRNA (for PD-L1 siRNA), and 0.2% DMSO (for PD98059) as 1.0. (*) represent the statistically significant difference (P < 0.05, N = 3, Student’s t-test) between the indicated samples and H_2_O, NT-siRNA, or 0.2% DMSO group.

(B)MDA-MB-453 were treated with PD98059 or DTT at the indicated concentration for 48h. DMSO and H_2_O are vehicles for PD98059 and DTT, respectively.


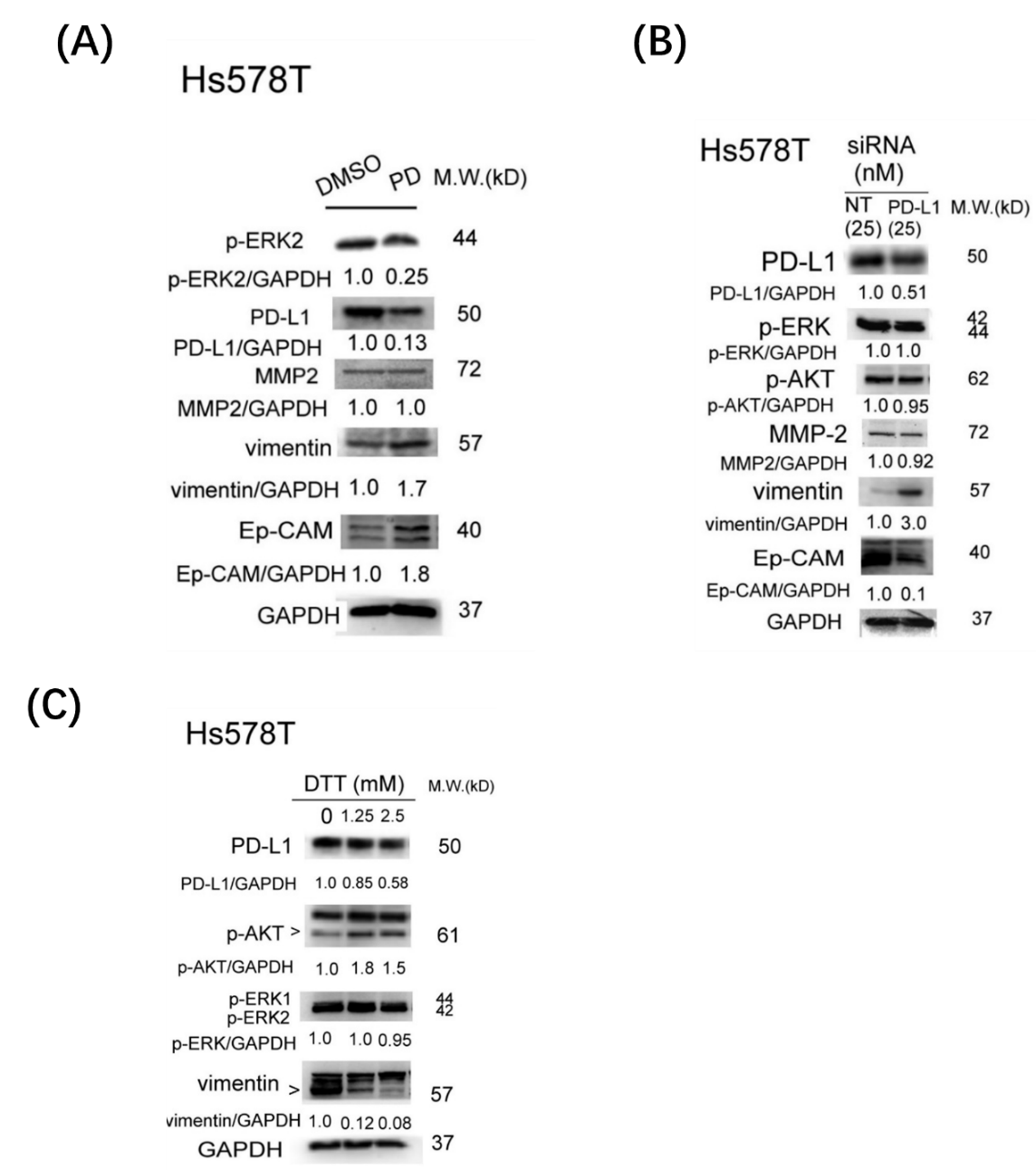


**Supplementary Fig. 5 Effects of PD98059, PD-L1siRNA, and DTT on signaling molecules and mesenchymal markers in Hs578T cells.**

(A) Hs578 T cells were treated with 40 μM PD98059 (PD) for 48 h;

(B) Hs578 T cells were transfected with PD-L1siRNA, and non-target (NT) siRNA at the indicated concentration;

(C) Hs578 T cells were treated with DTT at the indicated concentration for 48h.

In (A), (B) and (C), Western blots of the indicated molecules were performed using GAPDH as an internal control. The values below each band represent the relative intensities of the averages of three data of indicated molecules *vs* that of GAPDH, taking the DMSO (A), NT-siRNA (B) and DTT 0 (C) groups as 1.0
